# Splenic Marginal Zone Lymphoma and Concurrent Membranoproliferative Glomerulonephritis With IgMKappa Deposits in a HCV-Seropositive Patient

**DOI:** 10.5812/numonthly.18391

**Published:** 2014-07-05

**Authors:** Eleni Chelioti, Evdokia Efthimiou, Maria Sotiraki, Alexia Papalexandrou, Maria Tsilivigkou

**Affiliations:** 1 Department of Nephrology, General Hospital of Piraeus, Athens, Greece

**Keywords:** Hepatitis C, Membranoproliferative Glomerulonephritis, Lymphoma, B-Cell, Marginal Zone, Kidney, Biopsy

## Abstract

We report a case of membranoproliferative glomerulonephritis (MPGN) with IgMκ light chain deposits in a patient with chronic hepatitis C infection and simultaneous onset of monoclonal IgMκ gammopathy with concurrent small B-cell lymphoproliferative disease. The patient presented with hepatosplenomegaly and a uremic state that necessitated dialysis without any clinical signs of systemic disease apart from the chronic infection with hepatitis C virus. The diagnostic approach led to a renal biopsy that revealed MPGN with dominant IgMκ deposits and interstitium infiltration by the lymphoid cells. The bone marrow biopsy findings were consistent with splenic marginal zone lymphoma, a rare lymphoproliferative disorder with a rare association with MPGN. Our case indicates high diagnostic value of renal biopsy for rare lymphoplasmacytic neoplasms with renal dysfunction as their predominant clinical manifestation.

## 1. Introduction

Membranoproliferative glomerulonephritis (MPGN) is classified ast wo major groups: immune complex-mediated MPGN and complement-mediated MPGN; however, rarely, neither immunoglobulin nor complement deposition are detected in MPGN. Immune complex-mediated MPGN can be seen in chronic infections, autoimmune diseases, and monoclonal gammopathies (1). Chronic infection with Hepatitis C virus (HCV) can lead to several extrahepatic manifestations including MPGN, which is usually associated with essential mixed cryoglobulinemia and hematologic diseases such as cryoglobulinemia and lymphoma. On the other hand, non-Hodgkin lymphomas (NHLs) including splenic marginal zone lymphoma (SMZL) have been described as a rare cause of MPGN (2).

## 2. Case Presentation

A 50-year-old Caucasian male with a history of alcohol and injection-drug abuse, chronic HCV infection, coronary heart disease, and idiopathic renal function impairment (creatinine clearance < 60 mL/min) for preceding four months attended in the emergency department with uremic symptomatology. He was dysphonic (O_2 _saturation, 85%) and hypertensive (blood pressure, 185/90 mmHg) and had prominent hepatosplenomegaly with mild peripheral edema; he had no palpable lymph nodes, skin rash, or other manifestations of systemic diseases. Laboratory analyses results were as follows: metabolic acidosis (pH, 7.123; and HCO_3_^-^, 8.6 mmol/L); mild hyperkalemia (K^+^, 5.7 mmol/L); hyperphosphatemia (PO_4_^3-^, 10 mg/dL); hypoalbuminemia (albumin, 2.8g/L); anemia (hematocrit, 21.2%; and hemoglobin, 6.8 g/dL); thrombocytopenia (platelet, 72 × 10³/μL); lactate dehydrogenase,185 U/L; and severe renal failure (serum creatinine, 9.9 mg/dL; and urea, 199 mg/dL) with signs of glomerular involvement (urine red blood cells, 40 to 60/HPF; and 24-hour urine proteins, 3.06 g).

**Figure 1.**
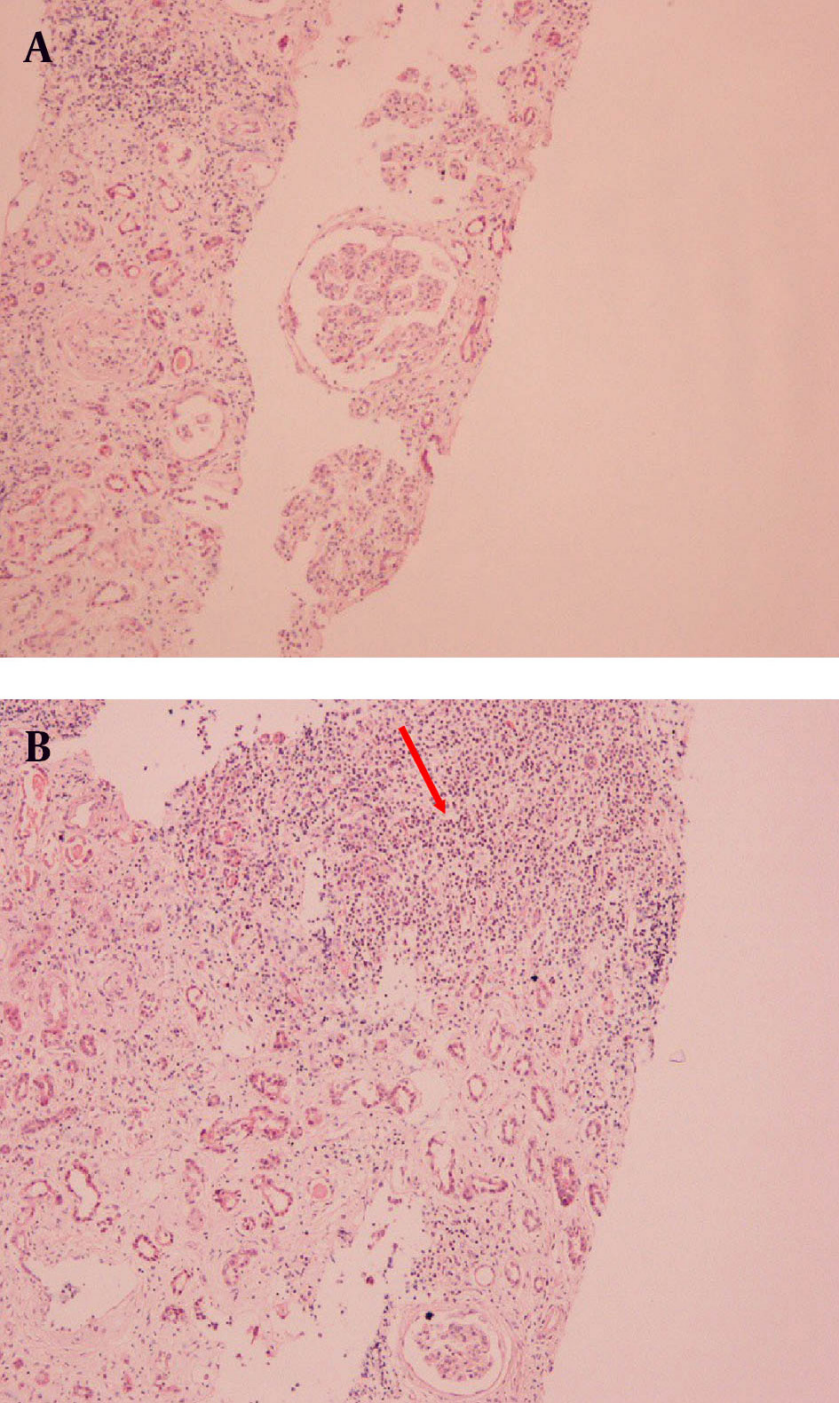
A, On light microscopy, lesions ofmembranoproliferative glomerulonephritis (MPGN), increase in lobular appearance of the glomerular tuft, and significant increase in cellularity are seen (HE × 100 magnification in A-B, Interstitium Infiltrated by strongly IgM-positive lymphoid cells (HE × 40 magnification in B). Abbeviation: HE,Hematoxylin, Eosin.

The clinical and laboratory findings on admission necessitated the immediate start of hemodialysis. Subsequent ultrasound studies revealed hepatosplenomegaly, two kidneys of normal size (right kidney, 11.7 cm; and left kidney, 11.5 cm), and parenchymal thickness (right kidney, 10 mm; and left kidney, 9.9 mm). There was no clinical or radiological sign of lymph nodes involvement. Further laboratory testing by immunological assay (ANA, anti-dsDNA, ASMA, AMA, C-ANCA, P-ANCA, and C3 complement levels) had negative results except for serum proteins electrophoresis, which showed a monoclonal gammopathy of IgM. At the same time, urine immunofixation revealed the presence of albumin, IgM, and kappa light chains, type I cryoglobulinemia, and low C4 complement levels. Considering the history of the patient and after excluding and treating all other causes of renal function deterioration, our diagnostic approach pointed towards an HCV-related renal offence in the context of cryoglobulinemia. However, the rapid decline of renal function and the absence of mixed cryoglobulinemia suggested performing the renal biopsy. While the histopathological examination of the specimen was consistent with the initial suspicion, indicating MPGN (Figure 1 A), the immunofluorescence study revealed two unusual findings: dominant monoclonal IgMκ deposits in glomerular basement membrane and infiltration of the interstitium by abundant strongly IgM-positive lymphoid cells (Figure 1 B). The presence of CD20 cell marker was distinctive while CD5 was absent. According to these findings, a bone marrow biopsy was performed. The histopathological examination revealed plasmacytic differentiation of small B cells and the immunophenotyping results were positive for CD20 and negative for CD5. These findings were consistent with SMZL. The patient became hemodialysis dependent and received no treatment for the lymphoproliferative disease and was monitored regularly for possible transformation to a high-differentiated lymphoma.

## 3. Discussion

The NHL subtype of marginal zone lymphoma includes three distinct diseases that have been historically classified together as they appear to arise from post germinal center marginal zone B cells and share a similar immunophenotyping findings including positive results for B-cell markers CD19, CD20, and CD22, and negative results for CD5, CD10, and usually CD23 (3). The three diseases in this category are splenic marginal zone B-cell lymphoma (SMZL), extranodal marginal zone B-cell lymphoma of mucosa-associated lymphoid tissue, and nodal marginal zone B-cell lymphoma. SMZL constitutes less than5% of all NHLs, 1% to 2% of indolent lymphoid leukemias found on bone marrow examination, and up to 25% of low grade B-cell neoplasms in splenectomy specimens. SMZL incidence has a median age of 65 years; the disease is uncommon before 50 years of age.

SMZL is postulated to arise from a post germinal centers, namely, splenic type memory B cells. The pathogenesis of disease is unclear. Epidemiologic studies have identified an association between SMZL and infection with some viruses such as HCV and Kaposi’s sarcoma-associated herpes virus. In the study of Hermine et al. (4), treatment of the HCV infection induced regression of SMZL in some patients; in addition, there was a correlation b tween lymphoma disease activity and HCV viral load. Since marginal zone lymphomas often arise in the setting of a sustained immunologic stimulus, it is possible that HCV antigens provide such a stimulus in HCV-associated cases. Patients typically present with splenomegaly, lymphocytosis, and cytopenias. Peripheral blood, focal bone marrow involvement, and liver infiltration are common. Rarely, patients will present with lymphocytosis as their sole marker of disease. Unlike most NHLs, lymphadenopathy and extralymphatic involvement are uncommon. In addition, approximately one-third of patients have a small amount of circulating monoclonal protein, often IgM. In those with HCV infection, mixed cryoglobulinemia and an associated small vessel vacuities may be present.

The diagnosis of SMZL is made by evaluating the lymphocyte morphology, immunophenotyping, cytogenetic analysis, bone marrow histopathology, and when available, spleen histology. If splenic tissue is not available for histopathologic examination, the diagnosis of SMZL can be made in a patient with clinical splenomegaly with typical morphologic and immunophenotyping findings on blood smear and bone marrow biopsy. The marrow usually contains discrete lymphoid aggregates, which may also have a marginal zone pattern, with or without diffuse intrasinusoidal lymphoid infiltration (5). The nodular pattern of involvement can be helpful in distinguishing SMZL from hairy cell leukemia, which always involves the marrow in a diffuse interstitial pattern. The immunophenotyping findings of SMZL is consistent with B-cell markers, i.e. CD19, CD20, and CD22 (6); typically, the immunophenotyping shows negative results for CD5, CD10, CD43 and CD25. Lack of CD5 serves to distinguish SMZL from chronic B-cell lymphocytic leukemia/small lymphocytic lymphoma and mantle cell lymphoma. Lack of CD103 and CD25 are useful in distinguishing SMZL from hairy cell leukemia.

The course of SMZL is generally extremely indolent, with a median overall survival of 10 years (7); however, some patients with a more aggressive course have a median survival of 18 months. Similar to other indolent lymphomas, SMZL has the potential to transform into a high-grade lymphoma (8). There are three possible prognostic factors of SMZL progression in the literature including hemoglobin of less than 12 g/dL, lactate dehydrogenase greater than normal level, and serum albumin level of less than 3.5 g/dL (9).

As a lymphoproliferative disorder, SMZL is rarely associated with MPGN (10). Immune complex-mediated MPGN is most commonly secondary to chronic hepatitis C or B viral infection. HCV-induced MPGN is typically associated with essential mixed cryoglobulinemia, defined by a mixture of polyclonal immunoglobulinsin association with a monoclonal immunoglobulins, typically IgM or IgA with rheumatoid factor activity (3). Type I cryoglobulinemia, characterized by the presence of isolated monoclonal immunoglobulins, typically IgG or IgM, and less commonly IgA or free immunoglobulin light chains, has not been identified in association with HCV-induced MPGN.

Chronic HCV infection has been associated with the development of B-cell NHL (including diffuse large B-cell lymphoma, marginal zone lymphoma, lymphoplasmacytic lymphoma, splenic lymphoma with villous lymphocytes, and extranodal marginal zone B-cell lymphoma of mucosa-associated lymphoid tissue). Glomerulonephritis is a rare complication of hematological malignancies. Chronic lymphocytic leukemia (CLL) and NHLs are the most common hematologic malignancies associated with glomerular diseases. MPGN is the most commonly reported CLL-associated glomerular disease, which is also associated with NHLs.

In our case, the predominant clinical features were those related to renal involvement. The patient had rapid deterioration of renal function (serum creatinine increased from 2.4 to 9.9 mg/dL within four months) without any other etiologic factors and absence of cryoglobulinemia or systemic disease symptomatology. Renal biopsy led diagnosis to a lymphoproliferative etiology and bone marrow biopsy revealed the SMZL. The patient joined a chronic hemodialysis program. Due to the indolent course of inactive HCV infection as well as SMZL, the patient was followed by a close hematological monitoring with regular testing of hemoglobin, lactate dehydrogenase, and albumin for possibility of transformation to a high-grade lymphoma.

We presented a rare case of SMZL as a cause of MPGN with IgMκ deposits and monoclonal gammopathy in an anti-HCV positive patient without mixed cryoglobulinemia. This case indicates that in case of renal disease manifestations, kidney biopsy can offer valuable information for diagnosis of rare lymphoproliferative disorders.
